# Antioxidant Effects of Statins by Modulating Nrf2 and Nrf2/HO-1 Signaling in Different Diseases

**DOI:** 10.3390/jcm11051313

**Published:** 2022-02-27

**Authors:** Atena Mansouri, Željko Reiner, Massimiliano Ruscica, Eugenia Tedeschi-Reiner, Shabnam Radbakhsh, Mariam Bagheri Ekta, Amirhossein Sahebkar

**Affiliations:** 1 Cellular and Molecular Research Center, Birjand University of Medical Sciences, Birjand 9717853577, Iran; mansouria1@yandex.com; 2 Biotechnology Research Center, Pharmaceutical Technology Institute, Mashhad University of Medical Sciences, Mashhad 9177948954, Iran; 3 Department of Internal Medicine, School of Medicine, University Hospital Center Zagreb, University of Zagreb, 10000 Zagreb, Croatia; zeljko.reiner@kbc-zagreb.hr; 4 Department of Pharmacological and Biomolecular Sciences, Università degli Studi di Milano, 20100 Milan, Italy; massimiliano.ruscica@unimi.it; 5 University Hospital Center Sestre Milosrdnice, University of Osijek, Vinogradska Cesta 29, 10000 Zagreb, Croatia; eugenia.tedeschireiner@gmail.com; 6 Student Research Committee, Mashhad University of Medical Sciences, Mashhad 9177948564, Iran; radbakhshs@yandex.com; 7 Department of Medical Biotechnology and Nanotechnology, Mashhad University of Medical Sciences, Mashhad 9177948564, Iran; 8 Laboratory of Cellular and Molecular Pathology of Cardiovascular System, A.P. Avtsyn Research Institute of Human Morphology, 3 Tsyurupy Str., 117418 Moscow, Russia; ektamb1@yandex.com; 9 Applied Biomedical Research Center, Mashhad University of Medical Sciences, Mashhad 9177948564, Iran; 10 Department of Biotechnology, School of Pharmacy, Mashhad University of Medical Sciences, Mashhad 9177948954, Iran

**Keywords:** statins, Nrf2, HO-1, antioxidants, anti-inflammation

## Abstract

Statins are competitive inhibitors of hydroxymethylglutaryl-CoA (HMG-CoA) reductase and have been used to treat elevated low-density lipoprotein cholesterol (LDL-C) for almost four decades. Antioxidant and anti-inflammatory properties which are independent of the lipid-lowering effects of statins, i.e., their pleiotropic effects, might be beneficial in the prevention or treatment of many diseases. This review discusses the antioxidant effects of statins achieved by modulating the nuclear factor erythroid 2 related factor 2/ heme oxygenase-1 (Nrf2/HO-1) pathway in different organs and diseases. Nrf2 and other proteins involved in the Nrf2/HO-1 signaling pathway have a crucial role in cellular responses to oxidative stress, which is a risk factor for ASCVD. Statins can significantly increase the DNA-binding activity of Nrf2 and induce the expression of its target genes, such as HO-1 and glutathione peroxidase) GPx, (thus protecting the cells against oxidative stress. Antioxidant and anti-inflammatory properties of statins, which are independent of their lipid-lowering effects, could be partly explained by the modulation of the Nrf2/HO-1 pathway.

## 1. Introduction

Statins are lipid-lowering drugs which inhibit the activity of the hydroxymethylglutaryl-CoA (HMG-CoA) reductase enzyme in the cholesterol synthesis pathway [1,2]. They have been widely used for the prevention and treatment of coronary artery disease (CAD) in hypercholesterolemic patients during almost the past four decades [3] and despite the introduction of newer agents [4,5,6,7]. Beyond their lipid-lowering activity, statins can modulate multiple metabolic pathways in different tissues and organs by which they achieve beneficial therapeutic effects in different diseases [8] Pravastatin (PRA), pitavastatin (PTV), rosuvastatin (RSV), lovastatin (LOV), simvastatin (SIM), atorvastatin (ATV), and fluvastatin (FLV) are different types of statins [9] that have not only low density lipoproteins cholesterol (LDL-C) lowering effects but many other pleiotropic beneficial effects such as anti-inflammatory, antioxidant, anti-apoptotic, and immunomodulatory [9,10,11,12,13,14,15]. Oxidative stress has an essential role in nitric oxide (NO) releasing, foam cell formation, atherosclerotic lesion progression, and instability of atherosclerotic plaque which are all involved in the process of atherogenesis [16,17]. Because of their effects on the antioxidant enzyme system, including lipoxygenases (LOXs), myeloperoxidase (MPO), nicotinamide adenine dinucleotide phosphate (NAD[P]H) oxidase, catalase, GPX, and superoxide dismutase (SOD) [18,19,20], statins can reduce atherosclerosis by their antioxidant effects. Girona et al. have demonstrated the antioxidant effect of SIM on lipoprotein particles in a preclinical study. SIM can decrease both oxidized HDL and oxidized LDL particles in a concentration-dependent manner [21]. Statins also achieve their antioxidant activity by their effects on nuclear factor erythroid 2-related factor 2 (Nrf2), a protein consisting of 589 amino acids with 66.1 kDa molecular mass, which belongs to the group of redox-sensitive transcription factors. This transcriptional factor expressed in several tissues such as the spleen, heart, kidney, and liver can improve the oxidative stress by regulating the expression of antioxidant enzymes, ROS detoxification, and can maintain redox homeostasis [22].

**Figure 1:**
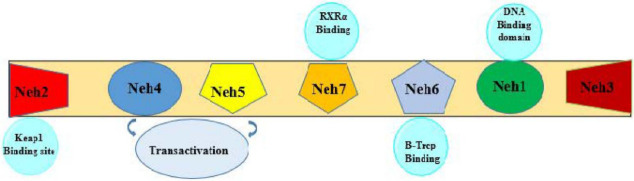
Conserved domains of Nrf2.

Nrf2 has seven NRF2-ECH homology (Neh) domains (Neh1–Neh7) (Figure 1) [22,23]. Three of them include Neh1, Neh3, and Neh6, and are located in the C-terminal while Neh2 is located in the N-terminal. Neh1 contains a leucine zipper motif which is important for DNA binding and dimerization with sMaf proteins. Neh2 is a negative regulatory domain and binds to cytoplasmic Kelch-like ECH-associated protein 1 (KEAP1). This domain has binding sites for DLG and ETGE motifs. Neh3, Neh4, and Neh5 domains are essential for the activation of transcription. Neh6 is a serine-rich conserved domain that targets Nrf2 for degradation.

The family of heme oxygenases (HO), which have three isoforms: *HO-1*, *HO-2,* and *HO-3* are important enzymes in heme catabolism and their function is to degrade it to free iron, carbon monoxide (CO), and biliverdin. *HO-2* and *HO-3* are expressed in cells and tissues. *HO-1* is inducible and responsible for Heme1 degradation. HO-1 is considered a cytoprotective enzyme because of its role in heme, biliverdin, and CO production. It has been shown that cell cultures and in vivo models have been protected because of its activity against oxidative stress, apoptosis, and eventual cell death [24,25,26,27,28].

## 2. Antioxidant Effects of Statins by Influencing Nrf2/HO-1 Pathway

Nrf2, as the main regulator, but also other proteins involved in the Nrf2/HO-1 signaling pathway, have a crucial role in cellular responses to oxidative stress [29]. Human cell cultures from HO-1 deficiency cases showed an increased sensitivity to oxidative injury, and HO-1 knockout animal models have more lipid peroxidation and higher levels of oxidized proteins suggesting antioxidant effects of HO-1 [30]. The Neh1 domain of Nrf2, together with the Maf protein, can bind to the antioxidant response element (ARE), which is a cis-regulatory module located in the promoter region of several antioxidant enzymes and cytoprotective proteins including catalase (CAT), superoxide dismutases (SOD), glutathione-S-transferase (GST), NAD(P)H-quinone oxidoreductase 1 (NQO1), thioredoxin reductase 1 (Txnrd1), and heme oxygenase-1 (HMOX1) [31,32]. In addition to the effects on antioxidant enzyme expression and activity, the Nrf2/HO-1 signaling axis can significantly attenuate ROS generation in mitochondria and modulate mitochondrial functional integrity [29].

Antioxidant and anti-inflammatory properties, which are independent of the lipid-lowering effects of statins, i.e., their pleiotropic effects, are involved in the protective mechanisms of these medicines which might be beneficial in the prevention or treatment of many diseases [33,34,35,36]. By regulating different oxidation pathways which control NADPH oxidase, myeloperoxidase, and endothelial nitric oxide synthase activity, statins promote antioxidation and restore redox homeostasis [37]. Besides their direct antioxidant effects, statins affect Nrf2/HO-1 signaling pathways causing the protection of cells against the detrimental effects of oxidative stress. Studies have shown that statins can significantly increase the DNA-binding activity of Nrf2 and induce the expression of its target genes, such as HO-1 and GPX, protecting the cells against the detrimental effects of oxidative stress [38]. Statin therapy also decreases reactive oxygen species (ROS) production by stimulating Nrf2 through the PI3K/Akt pathway [39]. It has to be mentioned that there are some data suggesting quite the opposite: that statins are able to cause toxicity based on oxidative stress in skeletal muscle, as well as neurotoxicity, hepatotoxicity, photosensitivity, and renal toxicity. However, in the following sections, we shall review the literature investigating the antioxidant effects of statins by modulating the Nrf2/HO-1 pathway in different organs and diseases (Table 1 and Table 2).

## 3. The Effects of Statins on Different Organs and Diseases

### 3.1. Effects on the Heart and Cardiovascular Diseases

Due to its high prevalence and burden, cardiovascular disease (CVD) remains a major global problem. According to the latest statistical information, 92.1 million adults living in the United States have one form of CVD. Almost 31.5% or 17.3 million people die because of CVD each year in the world. One of the most severe forms of CVD is myocardial infarction (MI), which affects more than 790,000 persons in the US each year [68,69]. However, there has been a consistent improvement in trends for long-term survival and outcomes after MI [70,71,72,73]. Increased reactive oxygen species (ROS) are the cause of the ischemic damage of myocardial cells in the early stages of MI. Myocytes are exposed to additional oxidative stress by reperfusion; however, this process is essential for maintaining the myocardial cells for life. Based on troponin concentration, the amount of myocardial cell death was measured and it has been established that ROS plays an important role in ischemia and cell death [72,74]. Innate defense mechanisms have been evaluated to identify the mechanisms, which might improve the situation after myocardial damage. Nrf2 has been identified as a protective substance in different tissues including the heart muscle. Because of the critical role of Nrf2 in multifaceted cellular defense, research efforts on this issue have increased in the recent periods. Nrf2 is activated by oxidative stress causing the expression of cytoprotective genes. Statins inhibit ROS production by concomitant suppression of Rac1/NADPH oxidase activity and up-regulation of the activity of GPx, thereby reducing VEGF expression [75]. Evidence shows that statins suppress the inflammatory reactions via the Nrf2 pathway. The treatment with atorvastatin achieved blood flow through the occluded left anterior descending artery (LAD), inducing HO-1 and Nrf2 and therefore reducing the infarction size. Besides lipid-lowering effects, the statins have other beneficial effects in atherosclerosis. The transcription factor Nrf2, under conditions of oxidative stress, activates the expression of HO-1, among other proteins. The Nrf2/HO-1 pathway is highly related to the initiation and development of atherosclerosis. It could be concluded that statins improve CVD by inducing an antioxidant HO-1 defense mechanism and remodeling of the myocardial structure [76], but also have anti-atherosclerotic effects apart from lipid-lowering [77].

### 3.2. Effects on Lung Diseases

The World Health Organization (WHO) reports that the third common cause of mortality is chronic respiratory diseases, including chronic obstructive pulmonary disease (COPD), cystic fibrosis, asthma, and bronchiectasis [78]. Despite the increased incidence of chronic pulmonary diseases, there is no substantially new and advanced treatment in the last years, at least not for some of them [79]. This increase could be explained by the increasing number of smokers and increased environmental pollution [78]. Of course, there are some established treatments for these diseases including anti-inflammatory drugs, β-adrenergic agonists, steroids, and antibiotics [75,80]. However, some patients are drug-resistant to these treatments and they need other effective treatments to replace them [81]. Some of the alternative drugs for chronic lung diseases might be statins. Although the main effect of statins is LDL-C lowering, these drugs also have cholesterol-independent effects, which might be beneficial in several diseases including chronic respiratory diseases. Both in vitro and in vivo studies have shown that MVS inhibited TNF’s ability to induce adhesion molecules by inducing the antioxidant enzyme (HO-1). Studies have demonstrated that MVS induced HO-1 expression is mediated by signaling components and transcription factors such as P47phox, Nox2, c-Src, PDGFR, Akt, and Nrf2. HO-1 expression against pulmonary inflammation was detected due to the p47phox-Nox2-dependent activation of PDGFR/PI3K/Akt and activation of the Nrf2/AREs pathways, which appeared to protect against inflammatory reactions [45]. Treatment with statins has many advantages including reducing the cost, risks, and toxicity. Studies have shown that statins have pleiotropic effects interfering with different pathways suppressing inflammatory, oxidative stress, and proliferation activities [82]. There were some data indicating that treatment with statins might be associated with statin-induced interstitial pneumonia [83]. However, some other studies have suggested that statins were associated with the reduced risk of idiopathic pulmonary fibrosis [84]. It has to be stated that at the moment there is not sufficient evidence to conclude that there is a clear deleterious or beneficial effect of statins on disease-related outcomes in idiopathic pulmonary fibrosis [85,86].

### 3.3. Effects on Kidney Disease

Chronic kidney disease (CKD) affects 11% of the adult population in the United States [87]. CKD has five stages based mostly upon glomerular filtration rate (GFR) [88,89]. Stages 1 and 2 have reduced kidney function but still normal GFR. Stages 3 to 5 have increasing reductions in GFR. The health condition of patients with CKD depends also on concomitant diseases, e.g., diabetes mellitus. These patients can have CVD and die before they reach end-stage renal disease. CKD is one of the important risk factors for CVD, especially in patients who are, because of other risk factors, at high risk for CVD [90]. Studies have shown that statins could reduce the severity of glomerular damage and preserve renal function [91,92,93]. New Zealand rabbits fed on a high cholesterol diet had glomerular hypertrophy and glomerulosclerosis. When these rabbits were treated with ATV, their plasma cholesterol was reduced and renal injury including glomerular hypertrophy was prevented. However, the mechanism by which statins may protect renal function is not clear. Statins inhibit HMG-CoA reductase which is important not only for endogenous cholesterol synthesis but they also inhibit geranyl pyrophosphate and isoprenoids as well as farnesyl pyrophosphate [94,95]. Isoprenoids cause different cellular responses like cell proliferation and migration as well as different gene expression by binding to intracellular signaling proteins.

Studies have shown that targeting oxidative stress can improve renal IR injury. An important pathway which is associated with antioxidative stress is nuclear factor erythroid 2 related factor 2 (Nrf2)/heme oxygenase 1 (HO1). The transcription factor Nrf2, a factor that can bind to antioxidant response elements (ARE), is located at the promoter regions of many antioxidant and detoxifying genes, including HO1. In addition, it seems that acetylcysteine has anti-inflammatory effects and improves renal injury by affecting Nrf2/HO1 activity [96]. Statins block the synthesis of isoprenoids and therefore they are able to restore the impaired capacity of progenitor cells. GFR is increased in oxidative stress. Renal injury is associated with elevated levels of inflammatory factors [97] and statins can reduce the inflammatory response. In a trial on 91 patients with CKD who were treated with 10 mg/day of rosuvastatin for 20 weeks, a 47% reduction in high specific C-reactive protein (hsCRP) and an improvement of GFR for 11% was achieved [98]. Although this indicates that statins might be effective in preventing CVD, more research is needed to be performed on patients with CKD to provide a clear answer about this issue [87].

### 3.4. Effects on Liver Disease

One of the important functions of the liver is cholesterol metabolism and the liver is also the main organ in which the effects of statins on cholesterol synthesis but also on plasma cholesterol levels occur. The risk of CVD is increased in liver diseases including hepatitis C virus (HCV) infection, primary biliary cirrhosis (PBC), and non-alcoholic fatty liver disease (NAFLD) [99,100,101]. Statins have beneficial effects in patients with chronic liver disease. Statins reduce cholesterol synthesis in the liver by regulating LDL receptors [102]. Statins have also pleiotropic effects that are not associated with HMG-CoA reductase inhibition, including antioxidant effects, anti-inflammatory effects, and effects on improving endothelial dysfunction [103]. Studies on NAFLD patients have shown that higher hsCRP is related with more severe NAFLD [104]. The activity of statins depends upon meta-substituents and lipophilicity of ortho-substituents on biphenyl and aryl moieties. Hepatic uptake transporter(s) play important roles in the clearance of different statins [105]. For example, some of them are metabolized by cytochrome P450 (fluvastatin, atorvastatin, lovastatin, and simvastatin). Other statins are metabolized in the liver (rosuvastatin, pitavastatin, and pravastatin) [103]. A meta-analysis showed that statins with lower lipophilicity and in higher doses have higher aminotransferase activity [106]. Nrf2 target genes HO-1 and GPX2 are enhanced by simvastatin. The livers of animals treated with simvastatin also showed Nrf2 activation. Because simvastatin had the same effect on Nrf2 in primary rat hepatocytes as it did in human cells, these effects were attributed directly to its action on the liver [107]. In addition to inhibiting the synthesis of cholesterol, statins also inhibit the formation of isoprenoids, like isopentenyl pyrophosphate and farnesyl pyrophosphate, by interfering with the production of mevalonate. Since the production of isoprenoids are inhibited, other important molecules are affected as well. Some of them are selenoproteins GPX1 and TRXR. The ability of statins to block the synthesis of selenoproteins GPX1 and TRXR may be one of the mechanisms by which they inhibit Nrf2 activation. GPX1 mRNA levels are decreased in the liver of selenium deficient rats. It has been recently demonstrated that Nrf2-mediated cytoprotective responses balance the reduced selenoprotein activity, which is necessary for maintaining cellular redox homeostasis. The reduction of both GPX1 and TRXR by simvastatin may contribute to the potentiation of Nrf2 and, consequently, the increased expression of GPX2. The response of selenoproteins to selenium deficiency seems to be different. Specifically, selenium deficiency increases GPX2 and decreases GPX1 in HepG2 cells [108] Although statins seem to be beneficial in patients with chronic liver disease, not much is known about the effects of statins on abnormal cell signaling pathways and liver histology; therefore, more studies on this issue are needed [109].

### 3.5. Effects on Eye Diseases

Statins have beneficial effects on many tissues including the eyes. However, there was a vivid debate in the past about the beneficial effects of statins on these diseases. In the early 1980s, physicians recommended that before starting statin therapy, eye exams should be made because of the alleged correlation with incident cataracts [110]. However, it has been shown that persistent statin use was significantly protective for the incidence of cataract in men and women under 75 years of age as well as for age-related macular degeneration (ARMD) [111]. More recent studies failed to prove the benefit of statins for ARMD. Actually, it has been shown that more than one year of statin use was associated with an increased hazard for exudative ARMD, but the authors of this study have clearly stated that their observations warrant further study and should not be the rationale for any changes in the use of statins to treat dyslipidemias [112]. Some studies discussed the relationship between statins and glaucoma progression, and their results showed that patients on statins did have less glaucoma progression. It has been shown that long-term use of statins seems to be associated with a reduced risk of open-angle glaucoma (OAG) [113,114]. About three million US citizens suffer from glaucoma [115]. In a study on 524 109 individuals with hyperlipidemia, the hazard of developing OAG decreased by 0.3% for every additional month of treatment with statins and those who were treated with statins continuously for two years had an 8% decreased OAG risk compared to those who received no statin therapy. They also had a 9% decreased risk of progressing OAG glaucoma when compared with those who received no statin therapy [113]. Oxidative stress affects the eye seriously. There are several oxidative conditions that contribute to the exposure to such stress including photooxidation, ionizing radiation, and smoke. Its high metabolism makes the retina one of the most perfused and oxygenated tissues in the body. Likewise, the content of polyunsaturated fatty acids is higher than those found in other body tissues. Consequently, it is vulnerable to oxidizing substances like ROS. Oxidative stress has been demonstrated to influence many ocular diseases such as diabetic retinopathy (DR), age-related macular degeneration (AMD), glaucoma, and cataract. The pathophysiology of the major ocular diseases includes inflammation and oxidative stress. Several studies have demonstrated that Nrf2 has both antioxidant and anti-inflammatory effects. The protection provided by medicines such as statins can be therefore beneficial [116]. The explanation why statins might have these effects could be that they are regulating nitric oxide synthase thereby increasing the blood flow to the optic nerve and retina [117]. Statins could also inhibit apoptosis in the central nervous system [118]. This could be an explanation for the beneficial effects of statins on these eye diseases.

### 3.6. Effects on Cancer

Despite the concern that statins might cause cancer, many studies have shown a preventive potential of statins concerning cancer [119,120]. The mevalonate pathway may be limited by HMG-CoA reductase and since statins are inhibitors of HMG-CoA reductase, they decrease mevalonate and products downstream of mevalonate. These substances are necessary for important cellular functions including cell signaling, protein synthesis, cell membrane integrity, and cell cycle progression [119]. Therefore, statins might control tumorigenesis and tumor metastasis by disrupting neoplastic cell pathways. Statins also suppress tumor activity by inhibiting matrix metalloproteinases (MMPs) [121]. MMPs have a role in anti-tumorigenesis because of degrading the extracellular matrix involved in the growth and invasion of tumors. According to some studies, statins decrease cell invasiveness and have beneficial effects on apoptosis [120]. Research on cell lines of mammary carcinoma [122,123], prostate carcinoma [124], colorectal carcinoma [125], lung carcinoma [126], and pancreatic carcinoma have supported these findings. However, the possible role of statins in the treatment of cancer still remains unclear and more studies are needed. There is no proof that statins are beneficial in breast cancer [127,128] although some studies reported a positive role of statins in this type of cancer. Cauley et al. showed a lower risk of breast cancer in patients treated with statins [129,130], but the results of six cohort studies [128,129,131,132,133,134] and two case-control studies could not support either positive or negative associations between statins and the occurrence of cancer [135,136]. The effect of statins on cell cycle progression may be annulled if statins were used with hormones, especially estrogens [132]. The results of a study on the association between statins and estrogen plus progestin showed a reduced risk of breast cancer [129]. This is important in the context of anti-cancer effects of statins in breast cancer cell lines, but the results of RCT are still insufficient. There are some phase II RCTs on statins and cancer, for example, exploring the effect of ATV on tumor proliferation, the effect of FLV on biomarkers, as well as the effect of SIM on the prevention during the early stage of breast cancer. Several studies analyzed the effect of statins on colorectal cancer. An important issue concerning colorectal cancer and breast cancer is that patients treated with statins when compared with healthy subjects are more likely to seek out preventive health services such as screening tests and have healthier lifestyles like performing regular exercise and sticking to the healthier diet [137,138]. The first RCT with ATV in phase II showed a decreased risk of colorectal neoplasia [137]. Another phase III trial with RSV and a phase II study showed beneficial effects of SIM in metastatic colorectal cancer patients. Many studies were performed on statins and lung cancer [139,140]. They have shown that statin treatment is associated with lower lung cancer risk and related mortality [86]. Studies were performed also on the efficacy of statins in prostate cancer prevention but to make definitive conclusions the epidemiologic studies should take into account the type of statins used and the serum concentrations achieved and ensure that the tested statin inhibits the specific type of cancer in vitro at those concentrations [141,142]. An important marker for prostate cancer is PSA. Studies have shown that statins reduce PSA levels [143] and serum PSA was significantly lower in patients with prostate cancer who were treated preoperatively with statins compared with those who were not taking statins [144,145]. However, the association between statin treatment and prostate cancer, similarly to the association with other types of cancer, needs further studies. HO-1 is expressed in cancer cells by nuclear translocation of Nrf2 by the ERK pathway and by the PI3K/Akt pathway, which is affected by simvastatin. In endothelial cells, statins activate HO-1 and decrease free radical production. These effects allow them to act as antioxidants and anti-inflammatory agents [146]. Carbon monoxide (CO) and free iron (Fe2) are then produced by the family of HO enzymes and heme is degraded to biliverdin. Bilirubin (derived from biliverdin degradation) and CO protect molecules from oxidative stress. Superoxide anions are reduced, lipid peroxidation is suppressed, apoptosis is prevented, vasodilation is increased, and local blood flow is improved. The expression of HO-1 can be high in some tumor cells and inhibiting the enzyme by specific inhibitors of this enzyme or by HO-1-shRNAs has been shown to inhibit the growth of some cancer cells. In contrast, HO-1 is known to display proapoptotic and anti-proliferative functions in cancer [147]. Simvastatin activates Nrf2, thereby up-regulating HO-1, NQO1, and GCLC expression in HT-29 and HCT116 cells. Nrf2 can promote tumor proliferation and prevent tumor development. ERK and PI3K/Akt signaling control the expression of Nrf2 and HO-1 in cancer cells. Simvastatin activates Nrf2 and increases its nuclear translocation in HT- 29 cells and stimulates the expression of antioxidants that are related to HO-1 via the ERK/PI3K/Akt pathway [66].

### 3.7. Effect on Neurodegenerative Disorders

The non-neuronal cells in the central nervous system (CNS) are glial cells or neuroglia, including microglia, oligodendroglia, and astroglia. These cells have an essential role in the metabolism of CNS [148,149]. Microglia are the primary innate immune system cells in the CNS [150]. They have also homeostatic regulatory properties [151]. Microgliosis is a strong reaction of microglia cells to pathogenic injuries [152]. Microglia activation causes the secretion of pro-inflammatory factors including cytokines, iNOS/NO, cyclooxygenase-2 (COX-2), and neuroinflammation [153]. The inflammation of the CNS is a defensive mechanism, and after the damage of the CNS, an immune response occurs. The inflammation causes the migration of immune system cells to the site of injury. Moreover, in the CNS, products of pro-inflammatory factors that are produced by activating microglia cells cause adverse effects on healthy neuronal cells and lead to neurological diseases (ND) such as Parkinson’s disease (PD), Alzheimer’s disease (AD), multiple sclerosis (MS), Huntington’s disease (HD), and others [154,155]. Statins have pleiotropic and anti-inflammatory effects [156,157]. Therefore, these drugs could be useful for protecting the CNS cells by their anti-neuroinflammation effects, they can improve angiogenesis, prevent superoxide free radicals, and improve phagocytic activity [158,159]. Recent research indicates that at least some statins inhibit microglia inflammation that induces mediators such as MMP-9, PGE2, IFN-γ, TNF-α, COX-2, and ROS/NO. Statins reduce the synthesis of cholesterol, and since hypercholesterolemia is an important risk factor for AD, they could improve AD. It seems that simvastatin can enhance neuroinflammation and oxidative stress response, reduce brain edema, improve neurological function, and improve the symptoms of cerebral hemorrhage. Statins reduce plasma cholesterol by inhibiting the mevalonate pathway. Mevalonate regulates the transduction of cellular signals and transcription factor activation [160]. After cerebral hemorrhage, inflammation factors such as IL-1, IL-6, and TNF-α can cause damage to brain tissues, causing neurological dysfunction. The expression of Nrf2 and HO-1 and NQO1 downstream molecules can be up-regulated in brain tissues, and they are increased after simvastatin administration. Simvastatin controls the Nrf2-ARE pathway regulating downstream signaling molecules and preventing oxidative stress. Furthermore, simvastatin can increase antioxidant enzyme SOD levels, but also reduce GSH and GSSG levels. Nerve damage occurs as a result of oxidative stress products, such as MDA and NO [161]. Statins can protect neurons from oxidative stress by up-regulating antioxidant enzyme expression. They also increase Nrf2 transcription. Simvastatin is thought to stimulate the Nrf2-ARE pathway through nucleic acid binding. This could provide a new therapeutic method for patients with cerebral hemorrhage since simvastatin could be used to enhance neurological function [162]. The pleiotropic effects of statins might also have a beneficial effect on the course of ischemic stroke. Statins could also activate microglia and have beneficial effects in neuroinflammation and neurodegenerative diseases [163].

### 3.8. Effect on Diabetes

Diabetes mellitus (DM) is one of the widespread diseases and its incidence is increasing in all parts of the world. The complications of DM are microvascular and macrovascular changes, the latter being an important cause of mortality [164]. The activation of Nrf2 in human and animal cells develops when a high concentration glucose stimulus is applied acutely. Alternatively, if glucose concentration oscillates or incubation times are longer, Nrf2 is not activated [165]. Nrf2 activation is also primarily determined by glucose concentration and cell type. Keap1-knockdown (KD) and Nrf2-knockout (KO) mice do not change insulin resistance since they lack the activity of the Nrf2/Keap1 systems. In diabetic kidney (based upon biopsy findings), in peripheral blood mononuclear cells (PBMCs), and skin tissue, Nrf2 seems to be an important factor in preventing micro- and macrovascular complications. Peripheral blood mononuclear cells (PBMC) obtained from prediabetic subjects have significantly reduced nuclear Nrf2. The down-regulation of Nrf2 during chronic hyperglycemia has been shown to be a potential treatment target. An important target for preventing diabetes mellitus is the Keap1-Nrf2 system [166]. Statins are some of the most important medicines which almost all patients with DM have to take [167]. They can prevent micro- and macrovascular changes and reduce cardiovascular risk in patients with DM [168]. Most of the patients with DM, even those with normal LDL-cholesterol levels, have to take statins and they have a proven beneficial effect even in these patients [169]. Statins improve endothelial dysfunction in diabetic patients and their beneficial effects may be explained in part by the attenuation of vascular O(2)(-) formation, which is independent of cholesterol lowering [170]. However, the protective mechanisms of statins have not been fully explained. Statins inhibit the production of L-mevalonic acid and prevent not only the synthesis of cholesterol but also the synthesis of isoprenoid intermediates. These intermediates such as farnesyl pyrophosphate (FPP) and geranylgeranyl pyrophosphate (GGPP) are essential in intracellular signal transduction. Small GTP-binding proteins, like Ras and Rho GTPase, are required for the normal structure and function of the cell membrane and statins have effects on a post-translational modification of small GTP-binding proteins. Therefore, statins might have beneficial effects in DM also by the inhibition of Rac-1 activity by reducing the availability of isoprenoid intermediates. It has been shown that long-lasting statin treatment is associated with a small but significant increase in incidence of new-onset type 2 diabetes [171,172]. However, this effect was limited to those who had impaired fasting glucose or multiple components of metabolic syndrome, i.e., those who were already at high risk of developing diabetes [173]. The mechanism of this adverse effect is not clearly understood but it seems that statins might interfere with insulin secretion in pancreatic beta cells, either by decreasing Ca-dependent insulin secretion or by interfering with guanosine triphosphate (GTP)-binding proteins [174].

## 4. Conclusions

Statins are drugs with LDL-C-lowering effects but also many pleiotropic effects [86]. Therefore, they might be useful in the treatment of many diseases, not only CVD but also cancer, kidney diseases, liver diseases, lung diseases, and neurodegenerative diseases. Statins have a good safety profile when used at recommended doses. Their adverse effects are rare and, like most drugs, occur with higher doses and prolonged treatment. The Nrf2/HO-1 signaling pathway is a protective antioxidative pathway that plays an important role in removing environmental and endogenous stressors; therefore, it is important in preventing the progression of different diseases. Statins have antioxidant and anti-inflammatory properties which are independent of their LDL-C-lowering effects. Namely, they can significantly increase the DNA-binding activity of Nrf2 and induce the expression of its target genes, such as HO-1 and GPX, thus protecting the cells against oxidative stress. These pleiotropic effects of statins are responsible for the protective mechanisms of these drugs involved in the prevention and/or treatment of several diseases which were already mentioned. By regulating oxidation pathways that control NADPH oxidase, myeloperoxidase, and endothelial nitric oxide synthase activity, statins promote antioxidant defense and restore redox homeostasis. However, much more data are needed to fully explain these effects of statins as well as more clinical trials to confirm the possible use of statins in the prevention and treatment of these diseases. It has to be stressed that statins, like other drugs, have also some adverse effects. The most prevalent ones are statin associated-muscle symptoms (SAMS) that can present as myalgia, myopathy, myositis with or without creatine kinase (CK) elevation, or in the most severe cases as rhabdomyolysis [175,176,177]. Statins slightly increase the risk of new-onset diabetes (9%) although the cardiovascular benefit outweighs the risk [178,179,180,181,182,183]. Early data indicated that statins raise transaminase. Nevertheless, more recent data suggest that statins can be even beneficial, at least in some liver diseases, e.g., non-alcoholic fatty liver disease (NAFLD) [184,185,186]. A similar situation was with the possible adverse effects of statins on kidneys. Earlier studies indicated possible adverse effects of statins on kidneys but more recent studies suggest that statin therapy in patients with chronic kidney disease (CKD) may even slow CKD progression [187,188]. Finally, in the elderly, the evaluation of the validity of a statin prescription should be based on a careful analysis of the patient’s general health, cardiac and peripheral artery evaluation, as well as the presence of metabolic abnormalities or drug interactions potentially leading to a risk of side effects, which are not rare in the very old [189].

## Figures and Tables

**Table 1:** Summary of in vivo studies investigating statin effect in various diseases.

Animal	Model	Diseases	Statin Type	Dose	Duration	Main Effect	Ref
Mice	Foxn1nu mice	Diabetes mellitus, cardiovascular diseases	Atorvastatin	30 μmol/L	2 weeks	Atorvastatin-treated precursors of myeloid angiogenic cells (PAC) — no effect on angiogenesis	[40]
Mice	BALB/c or C57BL/6 mice	Inflammatory diseases	Simvastatin and fluvastatin	5 mM simvastatin and 2 mM fluvastatin;	6, 12 or 24 h	Statins have anti-inflammatory effects and induce HO-1 in primary macrophages	[41]
Mice	male C57BL/6J mice	Coronary heart disease	Simvastatin	(0.75 to 5 mg/kg)	3, 6, 12, or 24 h	Simvastatin activates HO-1, HO-1 have cytoprotective effects, HO-1 are delivered to hearts and vessels of animal models in order to study myocardial protection—statins cause cardiovascular damage	[42]
Mice	male C57BL/6 mice	Chronic obstructive pulmonary disease (COPD)	Atorvastatin and simvastatin	1 mg/mL	60 days	Atorvastatin and simvastatin improve the repair of lung damage in mice exposed to cigarettes	[43]
	C57BL/6J female mice	Inflammatory lung diseases	Atorvastatin	5 mg/kg	10 days	Statins have anti-inflammatory effect by influencing HO-1 pathway in vivo	[44]
Mice		Inflammatory lung diseases	Mevastatin	0.1 mg/kg body weight	24 h	Mevastatin reduces TNF-α induced ICAM-1 expression via p47phox/Nox/ROS/c-Src/PDGFR_/PI3K/Akt/Nrf2/ARE/HO-1 - statins have beneficial effects in inflammatory lung diseases	[45]
Rats	(MCT-PH) and (CH-PH) rats	Pulmonary hypertension	Simvastatin	(10 mg/kgw	days 21~23)	Simvastatin decreases the severity of PH in two rat models (MCT- and CH-PH) by influencing HO-1 activity	[46]
Rat	Sprague-Dawley rats	Pulmonary hypertension	Simvastatin	2 mg/kg/day	4 weeks	Simvastatin therapy was useful in early phase of the pulmonary hypertension and in severe inflammation	[47]
Rat	Sprague-Dawley rats	Myocardial ischemia/reperfusion injury.	Atorvastatin	10 mg/kg, i.v		Atorvastatin has protective effects in myocardial ischemia reperfusion injury by activating Nrf2/ARE pathway	[48]
Mice	male C57BL/6 mice	Non-alcoholic steatohepatitis	Simvastatin	mg/kg	4 weeks	Simvastatin reduces liver damage caused by oxidative and endoplasmic reticulum stress effect in mice with experimental non-alcoholic steatohepatitis	[49]
Mice	C57BL/6J background	Cancers	Simvastatin	50 mg/kg	7 days	Due to their antioxidant effects statins might be effective in cancer treatment by influencing several specific metabolic pathways and increased oxidative stress which causes cancer effective in cure of cancer	[50]
Mice	C57BL/6J female mice	Inflammatory diseases	Atorvastatin	5 mg/kg	10 days	Statins have anti-inflammatory effect in inflammatory diseases due to their effect on HO-1 which is an anti-inflammatory substance	[44]
Mice		Pulmonary inflammation disease	Mevastatin	0.1 mg/kg	24 h	Mevastatin induces HO-1via c-Src/PDGFR_/PI3K/Akt-regulated Nrf2/ARE pathway—TNF-α is suppressed which finally improves inflammatory pulmonary disease	[45]
Mice	male C57BL/6J mice	Cardiovascular disease	Simvastatin	0.75 to 5 mg/kg	3, 6, 12, or 24 h	Simvastatin have anti-inflammatory and anti-proliferative effects in diseases by their influence on HO-1	[42]
Rat	adult male Sprague-Dawley rats	Heart disease	Simvastatin	2 mg/kg	1, 2 wk, 1, 2, and 3 mo	Simvastatin could not reduce inflammation and could not up-regulate HO-1	[51]

**Table 2:** Summary of in vitro studies investigating statin effect in various diseases.

Cell Culture	Diseases	Statin Type	Main Effect	Ref
Human RPE cells (ARPE-19; ATCC No.CRL-2302)	Age-related macular degeneration (ARMD)	Simvastatin	Simvastatin may have some clinical benefits in preventing ARMDs due to oxidative stress	[52]
Human HAoEC cells peripheral blood CD34+ cells	Diabetes mellitus, cardiovascular diseases	Atorvastatin	High concentrations of atorvastatin could improve the paracrine angiogenic activity	[40]
Murine RAW264.7 macrophages RAW264.7	Inflammatory diseases	Lovastatin, fluvastatin, atorvastatin, simvastatin, mevastatin, and pravastatin	Statins induce HO-1 gene expression and therefore have anti-inflammatory effects	[53]
Human pulmonary alveolar epithelial cells (HPAEpiC)	Inflammatory diseases	Mevastatin	Mevastatin induced HO-1 expression so it has an important role in inflammatory diseases via up-regulation of AP-1/HO-1 system	[54]
Primary human umbilical vein endothelial cells (HUVECs)	Cardiovascular diseases, chronic kidney disease	Rosuvastatin	Rosuvastatin has antioxidant effects by activating Nrf2 via p21Cip1 up-regulation - statins might be effective in improving antioxidative capacity	[55]
RAW 264.7 and J774A.1 cells	Inflammatory diseases	Simvastatin and fluvastatin	Statins have anti-inflammatory effects and induce HO-1 in macrophage cell lines	[41]
Rat aortic VSMCs (RASMCs) were isolated from thoracic aortas of Sprague-Dawley rats; human aortic VSMCs (HASMCs)	Coronary heart disease	Simvastatin	Simvastatin activates HO-1 which has cytoprotective effects - statins have anti-inflammatory and anti-proliferative effects	[42]
Mouse RAW264.7 macrophages	Hypercholesterolemia	Simvastatin	Simvastatin has anti-inflammatory effects due to induction of HO-1	[56]
HPAEpiCs	Inflammatory lung diseases	Mevastatin	Mevastatin reduces TNF-α induced ICAM-1 expression via p47phox/Nox/ROS/c-Src/PDGFR_/PI3K/Akt/Nrf2/ARE/HO-1—statins have anti-inflammatory effects in HPAEpiCs	[45]
Neuro-2A mouse neuroblastoma cells	Inflammatory diseases	Statins	Statins induce HO-1 by binding with p38	[51]
HT-29 cells HCT-116	Coloncancer	Simvastatin	Simvastatin improves colon cancer by activation of Nrf2 and expression of several antioxidant enzymes in pathways including ERK and PI3K/Akt	[47]
Vascular smooth muscle cells (VSMCs)	Diabetic vasculopathy	Fluvastatin	Statins improve diabetes complications by activating Nrf2 pathway reducing VSMC proliferation and migration and inducing AGEs and the ERK5-Nrf2 signal	[57]
Human coronary artery smooth muscle cells (hCASMCs)	Cardiovascular disease	Fluvastatin	Statins have a protective role in oxidative injury inducing antioxidant enzymes in Nrf2/ARE pathway	[58]
Human aortic smooth muscle cells (HASMCs)	Atherosclerosis	Atorvastatin	Simultaneous use of atorvastatin and C3G could activate Nrf2 pathway and increase antioxidative effects including GCLC, NQO-1, and HO-1 and SOD activity removing superoxide radicals and finally improving atherosclerosis	[59]
HL-1 cells	Atrial fibrillation (AF)	Rosuvastatin	Statins improve AF by activation of Akt/Nrf2/HO-1 signaling and inducing antioxidant HO-1	[60]
Human prostate adenocarcinoma (PC-3) and breast adenocarcinoma MCF-7 cell lines	Adenocarcinoma of the prostate and breast adenocarcinoma	Atorvastatin	Significant up-regulation of HO-1 (all six ARE-like elements are present in the HO-1 promotor activated by atorvastatin) and apoptosis was induced in both PC-3(at a concentration of 1 µM) and MCF-7(at a concentration of 50 µM) cell lines	[61]
Human umbilical endothelial cells HUVEC	Cardiovascular disease	Atorvastatin	Atorvastatin moderately increased eNOS and HO-1 mRNA expression but HO-1 protein levels did not change significantly	[62]
Human microvascular endothelial cells (HMEC-1)	Cardiovascular diseases	Atorvastatin	Atorvastatin at concentration 0.1 μM enhanced the expression of eNOS and was ineffective in modulation of HO-1 protein level	[63]
(NRK52E) cells	Lipotoxic injury in kidney	Simvastatin	Simvastatin transcriptionally activates HO-1 that protect renal cells from lipotoxic injury	[64]
SH-SY5Y cells	Parkinson’s disease	Simvastatin	Simvastatin has antioxidant activity via ERK1/2-mediated modulation of the antioxidant system	[65]
The rat renal proximal tubule cell line (NRK52E)	Kidney disease	Simvastatin	Antioxidant effect of simvastatin via HO-1 may protect the kidney	[64]
SH-SY5Y cells	Parkinson’s disease	Simvastatin	Simvastatin has antioxidant effect via ERK1/2-mediated reduction – this may decrease the incidence of Parkinson’s disease	[65]
Human endothelial cells cell line (ECV304)	Cardiovascular disease	Statins	Statins have antioxidant and anti-inflammatory effects via HMG-CoA reductase by activating HO-1 promoter	[66]
Murine RAW264.7	Inflammatory disease	Statins	Statins activate protein kinase A and after affecting ERK and p38 MAPK pathways finally activate HO-1 gene expression and acting on this pathway are beneficial in anti-inflammatory diseases	[53]
Rat aortic VSMCs (RASMCs)	Cardiovascular disease	Simvastatin	Simvastatin has anti-inflammatory and anti-proliferative effects by affecting HO-1	[42]
HCT116 and HT-29 cells	Colon cancer	Simvastatin	Simvastatin had beneficial effects in colon cancer cells by influencing Nrf2 and ERK and PI3K/Akt pathways	[51]
A mouse neuroblastoma cell line	Degenerative neurological diseases	Simvastatin	Simvastatin induced HO-1 expression in Neuro 2A cells by having effect on Nrf2 protein	[67]

## Data Availability

Raw data associated with this study are available from the corresponding authors upon a reasonable request.
